# Administration of allogeneic mesenchymal stem cells in lengthening phase accelerates early bone consolidation in rat distraction osteogenesis model

**DOI:** 10.1186/s13287-020-01635-5

**Published:** 2020-03-20

**Authors:** Yanhua Yang, Qi Pan, Kaijie Zou, Haixing Wang, Xiaoting Zhang, Zhengmeng Yang, Wayne Yuk Wai Lee, Bo Wei, Weidong Gu, Yunzhi Peter Yang, Sien Lin, Gang Li

**Affiliations:** 1 Department of Central Laboratory, Changzhou Seventh People’s Hospital, Changzhou, China; 2 Department of Orthopaedic and Traumatology, Faculty of Medicine, The Chinese University of Hong Kong, Hong Kong, China; 3 Stem Cells and Regenerative Medicine Laboratory, Li Ka Shing Institute of Health Sciences, The Chinese University of Hong Kong, Prince of Wales Hospital, Shatin, Hong Kong, China; 4 Orthopaedic Center, Affiliated Hospital of Guangdong Medical University, Guangdong Medical University, Zhanjiang, China; 5 Department of Orthopaedic Surgery, School of Medicine, Stanford University, Stanford, USA; 6 Department of Materials Science and Engineering, School of Engineering, Stanford University, Stanford, USA; 7 Department of Bioengineering, School of Medicine, Stanford University, Stanford, USA; 8 The CUHK-ACC Space Medicine Centre on Health Maintenance of Musculoskeletal System, The Chinese University of Hong Kong Shenzhen Research Institute, Shenzhen, China; 9 Key Laboratory for Regenerative Medicine, Ministry of Education, School of Biomedical Sciences, Faculty of Medicine, The Chinese University of Hong Kong, Hong Kong SAR, China

**Keywords:** Distraction osteogenesis, Mesenchymal stem cells, Bone consolidation, Cytokines

## Abstract

**Background:**

Distraction osteogenesis (DO) is a surgical technique to promote bone regeneration which may require long duration for bone consolidation. Bone marrow-derived mesenchymal stem cells (MSCs) have been applied to accelerate bone formation in DO. However, the optimal time point for cell therapy in DO remains unknown. This study sought to determine the optimal time point of cell administration to achieve early bone consolidation in DO. We hypothesized that the ratio of circulating MSCs to peripheral mononuclear cells and the level of cytokines in serum might be indicators for cell administration in DO.

**Methods:**

Unilateral tibial osteotomy with an external fixator was performed in adult Sprague Dawley rats. Three days after osteotomy, the tibia was lengthened at 0.5 mm/12 h for 5 days. At first, 5 rats were used to analyze the blood components at 6 different time points (3 days before lengthening, on the day lengthening began, or 3, 6, 10, or 14 days after lengthening began) by sorting circulating MSCs and measuring serum levels of stromal cell-derived factor 1 (SDF-1) and interleukin 1β. Then, 40 rats were used for cell therapy study. A single dose of 5 × 10^5^ allogeneic MSCs was locally injected at the lengthening site on day 3, 6, or 10 after lengthening began, or 3 doses of MSCs were injected at the three time points. Sequential X-ray radiographs were taken weekly. Endpoint examinations included micro-computed tomography analysis, mechanical testing, histomorphometry, and histology.

**Results:**

The number of circulating MSCs and serum level of SDF-1 were significantly increased during lengthening, and then decreased afterwards. Single injection of MSCs during lengthening phase (on day 3, but not day 6 or 10) significantly increased bone volume fraction, mechanical maximum loading, and bone mineralization of the regenerate. Triple injections of MSCs at three time points also significantly increased bone volume and maximum loading of the regenerates.

**Conclusion:**

This study demonstrated that bone consolidation could be accelerated by a single injection of MSCs during lengthening when the ratio of peripheral MSCs to mononuclear cells and the serum SDF-1 presented at peak levels concurrently, suggesting that day 3 after lengthening began may be the optimal time point for cell therapy to promote early bone consolidation.

## Background

Distraction osteogenesis (DO) is a surgical technique that applies tension stretching force on the bone and other surrounding tissues to stimulate bone and skeletal tissues regeneration [1]. DO has been widely used for the treatment of limb discrepancy, bone defect, nonunion, infection, and malformation, owing to its effectiveness [2–5]. Nevertheless, long treatment duration for bone consolidation in the distraction regenerate is a major limitation for clinical application of DO technique. Patients undergoing DO treatment must wear external fixator for a longer duration [6]. Long period of physical inconvenience and psychosocial burden are challenging for patients, and the probability of complications will also arise with prolonged time of wearing the external fixators [4, 7]. Therefore, there is a great need in accelerating bone consolidation in DO process.

Various attempts, such as biophysical stimulation including pulsed electromagnetic field, low-intensity pulsed ultrasound stimulation, or drugs including growth hormone and growth factors, have been tried to accelerate callus formation and shorten the consolidation period in DO with limited success [8]. Mesenchymal stem cell (MSC) transplantation is believed to be a promising regenerative therapy. MSCs have special characteristics in response to traumatic injuries and can produce regenerative cytokines, replicate themselves, and differentiate into specialized cell types of the tissue or organ. MSCs are essential for bone fracture healing, which can differentiate into chondrocytes, fibroblasts, or osteoblasts to form a fracture callus [9, 10]. Some positive findings showed that administration of MSCs in DO model has improved the quantity of bone formation and consolidation [10, 11]. However, several studies reported negative results on applying MSCs in DO [2, 4]. The inconsistency of these reports could be partially due to the different therapeutic time points [11], as the molecular response during the three phases of DO, including latency, lengthening, or consolidation phases, is different [12]. Regarding the therapeutic outcome of stem cell therapy in DO, there is still a lack of investigation to address the optimal time point. Our previous study has revealed the important role of circulating stem cells in promoting fracture healing [13, 14]. The recruitment of circulating MSCs in DO may be also helpful for the bone healing. We hypothesized that the level of circulating MSCs or cytokines during DO might be an indicator of the optimal timing for MSCs administration. In the current study, we firstly examine the dynamic changes of circulating MSCs and cytokines in a DO rat model. Secondly, based on these results, we further investigated the timing effect of administrating MSCs on bone consolidation in DO.

## Methods

### Animals and study design

Forty-five 20-week-old male Sprague Dawley (SD) rats were used. Animals were acclimatized to local vivarium conditions at temperature of 24–26 °C and a humidity of 70% with free access to water and a pelleted commercial diet. Animal experiments were carried out in accordance with Animal (Control of Experiments) Ordinance of Hong Kong Special Administrative Region (SAR) and approved by the Animal Experimental Ethical Committee (AEEC) of the Chinese University of Hong Kong. All surgeries were performed under anesthesia, and efforts were made to minimize the suffering of the animals. The animal study was divided into two parts. The first part was designed to determine the quantity of circulating MSCs and cytokines in the peripheral blood of the rats at different phases of DO. The second part was to evaluate the therapeutic effect of allogeneic MSCs on bone healing in DO. The researchers involved in the study were blinded during allocation, animal handling, and endpoint measurements.

### Animal surgery and DO protocol

Before surgery, each rat was anesthetized with a solution of 0.2% (v/v) xylazine and 1% (v/v) ketamine in PBS. All animals were subjected to a right tibia transverse osteotomy procedure at the midshaft near the fibula-tibia junction by low-speed dental driller under sterile condition as previously described in publications [15, 16]. Of note, the periosteum of the tibia should be retained as much as possible. A customer made monolateral external distraction fixator (Tianjing Xinzhong Co., Tianjin, China) was placed to fix proximal and distal segments of the osteotomy site. Surgical incisions were then sutured sequentially. The DO protocol consisted of three phases according to our previous reports [15, 16]: a latency phase of 5 days, a 5-day active lengthening phase (0.5 mm/12 h), and a consolidation phase of 28 days.

### Peripheral blood for flow cytometry and biochemistry assays

For the first part of the study, animals (*n* = 5) after DO surgery were used to collect blood to determine the ratio of circulating MSCs and cytokines in the peripheral blood. Three percent of isoflurane was used for inhalation anesthesia before blood collection. One milliliter of blood was harvested each time from the left or right retro-orbital of the rats, alternatively, 3 days before lengthening (day − 3), immediately when the lengthening began (day 0), and on day 3, 6, 10, or 14 after lengthening began.

Flow cytometry was performed after lysis of the red blood cells to identify circulating mesenchymal stem cells from the mononuclear cells by the surface markers of CD31, CD45, CD44, and CD90 (BD Biosciences, USA) as described previously [17]. The lineage differentiation potential of the CD31-CD45-CD44+CD90+ cells was determined by Alizarin Red S stain for osteogenesis, Oil Red O stain for adipogenesis, or Toluidine Blue stain for chondrogenesis as previously described [18]. MSCs were identified in the blood as CD31-CD45-CD44+CD90+ cells by BD FACS cell sorter (BD Biosciences, USA). ELISA was performed to test the expression level of stromal cell-derived factor 1 (SDF-1, Novus Bio, Centennial, CO, USA) and interleukin 1β (IL-1β, Boster, Pleasanton, CA, USA) according to the protocols attached in the commercial kits.

### Bone marrow-derived stromal cells culture

Adult male outbred green fluorescent protein (GFP) SD rats (SD-Tg (CAG-EGFP) Cz-004Osb) were used to isolate MSCs from the bone marrow and characterized by flow cytometry and lineage differentiation assays in this study, as previously reported [17, 19]. After that, the cells were regarded as mesenchymal stem cells (MSCs) and then cultured in modified Eagle’s medium of Alpha (α-MEM; Invitrogen, USA) supplemented with 10% fetal bovine serum (FBS; Gibco, USA) and 1% penicillin-streptomycin (PS) antibiotic mixture (Gibco, USA) at 37 °C with 5% CO_2_ and 95% humidity. The culture medium was changed every 3 days. The MSCs from passages 3 to 6 were used in the animal experiments.

### Stem cell therapy

The second part of this study was to evaluate the therapeutic effect of allogeneic MSCs on bone healing in DO. After surgery, forty SD rats were randomly assigned into 5 groups. The rats were administered with single dose of MSCs (5 × 10^5^ cells in 100 μl PBS) on day 3 (D3, *n* = 8), day 6 (D6, *n* = 8), or day 10 (D10, *n* = 8) after bone the lengthening began (day 0) or administered with single dose of PBS (100 μl) on day 3 after the lengthening began as blank controls (CON, *n* = 8). Another eight rats were injected with three doses of MSCs (5 × 10^5^ cells in 100 μl PBS for each dose), with one dose on day 3, day 6, and day 10 after the lengthening began, respectively (triple, *n* = 8). The dose of MSCs was chosen according to our previous study [11]. Before injections, MSCs were trypsinized and washed with PBS. The PBS with or without cells were injected immediately into the lengthening gap once the cells were prepared. To avoid any leakage, the injections were performed by gently inserting a 32-G needle into the defect side with the depth around 10 mm and waited for 5 s before slowly withdrawing the needle. The procedures were performed under anesthesia by isoflurane.

All rats received subcutaneous injection of calcein (10 mg/kg; Sigma-Aldrich, St. Louis, MO, USA) 13 days before termination and xylenol orange (30 mg/kg, Sigma-Aldrich, St. Louis, MO, USA) 3 days before termination for in vivo labeling. X-ray images were taken weekly to monitor bone healing. Thirty-three days after the lengthening began, the animals were terminated. Bilateral tibias were harvested and processed for further examinations. In order to follow the 3Rs (Replacement, Reduction and Refinement) principles for animal experiments, we performed micro-CT scanning first, followed by mechanical testing and histological analysis using the same specimens. Micro-CT and mechanical testing analysis were performed on the same day; the specimens were kept on ice and then fixed in 10% formalin immediately after mechanical tests.

### Micro-computed tomography (CT) analysis

Microstructural change within the distraction regenerate in the rat was quantitatively assessed using micro-CT as previously described [15]. Briefly, all the specimens were imaged using a high-solution micro-CT (Scanco Medical, Bassersdorf, Switzerland) at a custom isotropic resolution of 8 μm isometric voxel size with a voltage of 70 kV and a current of 114 μA. Three-dimensional (3D) reconstructions of the mineralized callus were performed using a global threshold (158 mg hydroxyapatite/cm^3^), and a Gaussian filter (sigma = 0.8, support = 2) was applied to suppress noise. Cross-sectional images of the distraction zone were used to perform 3D reconstruction analysis. The region of interest was defined as the distraction zone (regenerate) between the two closest proximal and distal half-pins. Bone mineral density (BMD) and bone volume/total tissue volume (BV/TV) of each specimen were recorded with the built-in software for analysis.

### Mechanical test

After micro-CT analysis, mechanical properties of specimens were evaluated by four-point bending test within 24 h after termination. A material testing system (H25KS; Hounsfield Test Equipment Ltd., UK) with a 250-N load cell was used to test the tibia to failure. The tibias were loaded in the anterior-posterior direction with the inner and outer span of the blades set as 8 and 18 mm, respectively. The bones were tested at a speed of 0.01 mm/s, with the long axis of the tibia placed perpendicular to the blades during the test. The modulus of elasticity (E-modulus), ultimate load, and energy to failure were obtained and analyzed with built-in software (QMAT Professional; Tinius Olsen, Inc., Horsham, PA, USA). The biomechanical properties of the new bone were expressed as percentages of the contralateral intact bone properties. During the mechanical tests, we stopped the compression once the loading showed a 15% decrease to make sure not to break the bone.

### Histology and immunohistochemistry in decalcified tissue

Immediately after mechanical tests, the specimens were initially fixed in 10% formalin for 48 h, and then transferred to 70% ethanol. All the tibias were cut sagittal into two equal parts by precision bone saw (Buehler, Lake Bluff, IL, USA). Half of the specimens were decalcified in 10% EDTA solution for 3 weeks and embedded into paraffin. Thin sections (5 μm) were cut by a rotary microtome (HM 355S, Thermo Fisher Scientific, Inc., Germany) along the long axis of each tibia in the sagittal plane. After deparaffinization, hematoxylin and eosin (H&E) staining and Safranin O & Fast Green staining were performed. The cartilage, fibrous tissue, or bone would be stained in red, white to light green, or green, respectively. The ratio of unmineralized tissue in the regenerate (cartilage and fibrous tissue in regenerate tissue/regenerate tissue, %) was determined by the Safranin O & Fast Green staining (Sigma-Aldrich, St Louis, MO) and analyzed Image-pro plus software (Media Cybernetic, Rockville, MD).

Immunohistochemistry staining was performed using a standard protocol. Samples were incubated with anti-osteocalcin (anti-OCN; Santa Cruz, Dallas, TX) or anti-green fluorescence protein antibody (anti-GFP; Santa Cruz, Dallas, TX) overnight at 4 °C. A horseradish peroxidase-streptavidin detection system (Dako, Santa Clara, CA) was used, followed by counterstaining with hematoxylin.

The positive stained tissue area in the whole distraction regenerate per specimen in two sequential sections (100 μm, and 200 μm) per rat in each group were counted, compared, and expressed as the percentage (*n* = 8).

### Histomorphometry in non-decalcified tissue

The other half of the specimens were taken through gradient alcohol dehydration, xylene defatting, and then embedded in methyl methacrylate. Ten-micrometer sections were cut with the RM2155 hard tissue microtome (Leica, Wetzlar, Germany) along the long axis of the tibia for non-decalcified tissue histomorphometric measurements. The abbreviations of the bone histomorphometric parameters used were recommended by the ASBMR Histomorphometric Nomenclature Committee [20]. All measured thicknesses were multiplied by π/4. The structural parameters are tissue volume (TV) and bone volume (BV). And the dynamic parameters are bone single-labeled surface (sL.S), double-labeled surface (dL.S), ratio of mineralizing surface to bone surface (MS/BS, calculated as double plus half of single-labeled surfaces (sL.S)), mineral apposition rate (MAR), bone formation rate per unit of bone surface (BFR/BS), and bone formation rate of bone volume (BFR/BV).

### Statistical analysis

All the quantitative data were presented as mean and standard deviation (SD). After checking of normal distribution by Kolmogorov-Smirnov test, all parameters were analyzed by ANOVA and post hoc Turkey’s HSD. For mechanical test, contralateral tibias were used to normalize the mechanical parameters. The statistical analysis was calculated by SPSS (version 16.0; SPSS Inc., Chicago, IL), and the level of significance was set at *p* < 0.05.

## Results

### Dynamic changes of circulating MSCs and cytokines

**Fig. 1:**
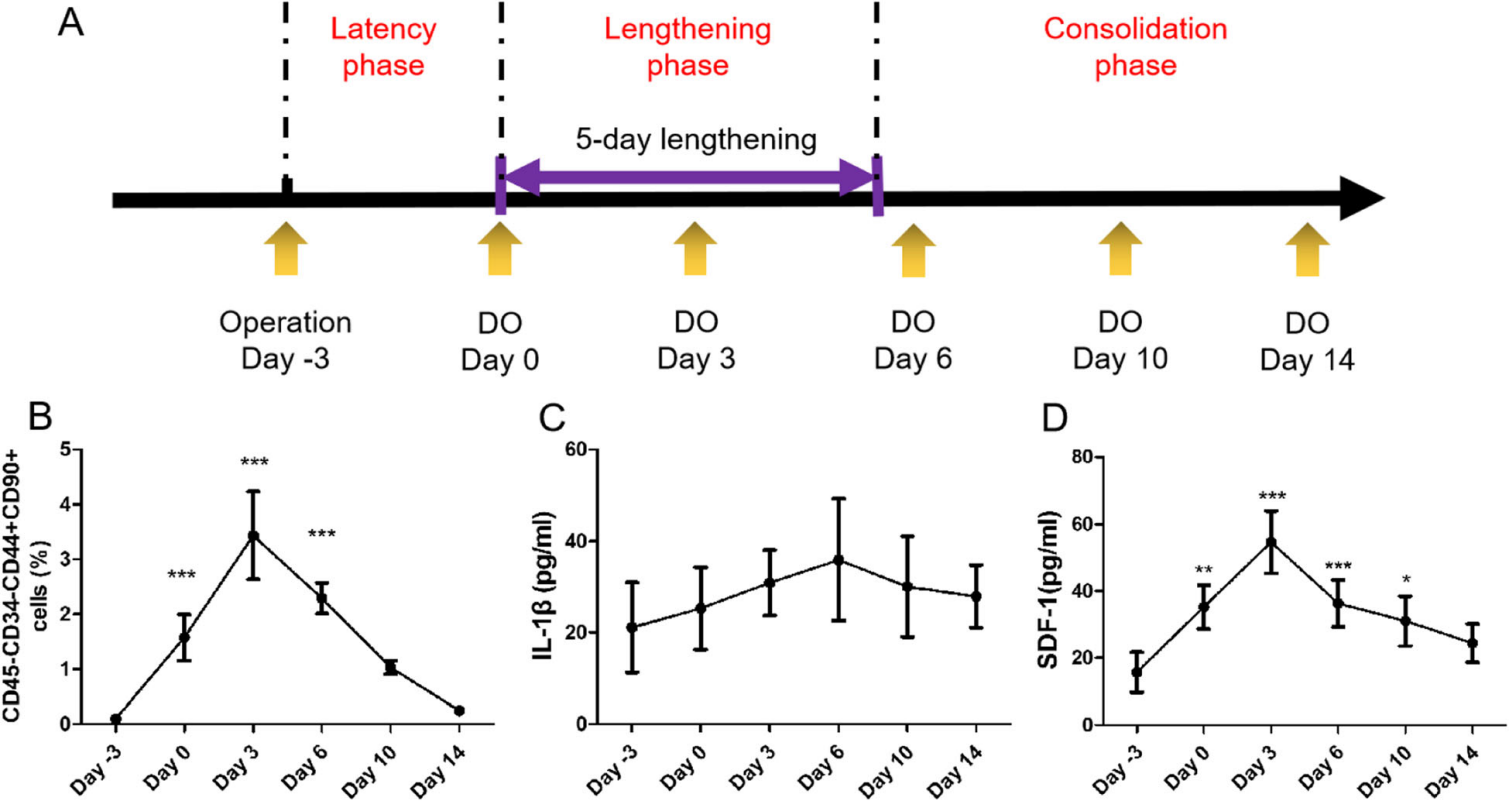
Timeline of blood harvesting time points and the quantity of circulating MSCs and cytokines in the peripheral blood in the DO animals. Blood was harvested on 3 days before bone lengthening (day − 3), the day of lengthening started (day 0), or 3 (day 3), 6 (day 6), 10 (day 10), or 14 (day 14) days after lengthening began. **a** Schematic timeline of peripheral blood harvesting time points in the DO animal model. **b** The ratio of CD31-CD45-CD44+CD90+ cells in mononuclear cells measured by flow cytometry. **c** Serum levels of IL-1β measured by ELISA. **d** Serum levels of SDF-1 measured by ELISA. Data were shown as mean ± SD (*n* = 5). *P < 0.05, **P < 0.01, ***P < 0.001 vs. (day − 3 group)

As illustrated in Fig. 1a, the timeline of DO experiment was divided into 3 phases, including latency phase, lengthening phase and consolidation phase. Blood was collected from left or right retro-orbital of the rats alternatively, 3 days before lengthening (day − 3), immediately when lengthening began (day 0), and on day 3, 6, 10, or 14 after lengthening began (Fig. 1a). To identify the circulating MSCs during DO, flow cytometry assays were performed at different time points of DO. The results showed that there was only 0.09% of the mononuclear cells that were CD31-CD45-CD44+CD90+ cells on the day of surgery (3 days before distraction, day − 3) (Fig. 1b). Interestingly, the ratio (1.57%, *p* < 0.001) of CD31-CD45-CD44+CD90+ cells to all mononuclear cells was significantly increased from the date of lengthening started (day 0) (Fig. 1b). Then, it reached a peak (3.44%, *p* < 0.001) 3 days after lengthening began (day 3) (Fig. 1b). However, the ratio (2.29%, *p* < 0.001) was gradually reduced from 6 days after lengthening began, and then returned to the normal level after 14 days of lengthening (Fig. 1b). The flow cytometry results indicated dynamic changes in the ratio of circulating MSCs during DO.

IL-1β is a pro-inflammatory marker, whereas SDF-1 is an important chemokine for MSCs migration. ELISA was conducted to determine the global expression levels of SDF-1 and IL-1β during DO. The results showed that the levels of IL-1β were not significantly changed during DO, except for an increasing trend in the lengthening phase (Fig. 1c). Interestingly, we found that serum levels of SDF-1 was greatly increased by 124.1% (*p* < 0.01), 247.8% (*p* < 0.001), 130.9% (*p* < 0.001), and 97.5% (*p* < 0.05) on days 0, 3, 6, and 10 after bone lengthening (Fig. 1d). The serum levels were gradually increased from day − 3 and reached to a peak on day 3 after lengthening began (Fig. 1d). And then, it gradually decreased afterwards and finally returned to normal on day 14 (Fig. 1d). The ELISA results indicated that the global expression level of SDF-1 was consistent with the dynamic changes in the ratio of circulating MSCs.

### Bone regeneration monitored by sequential X-ray imaging

**Fig. 2:**
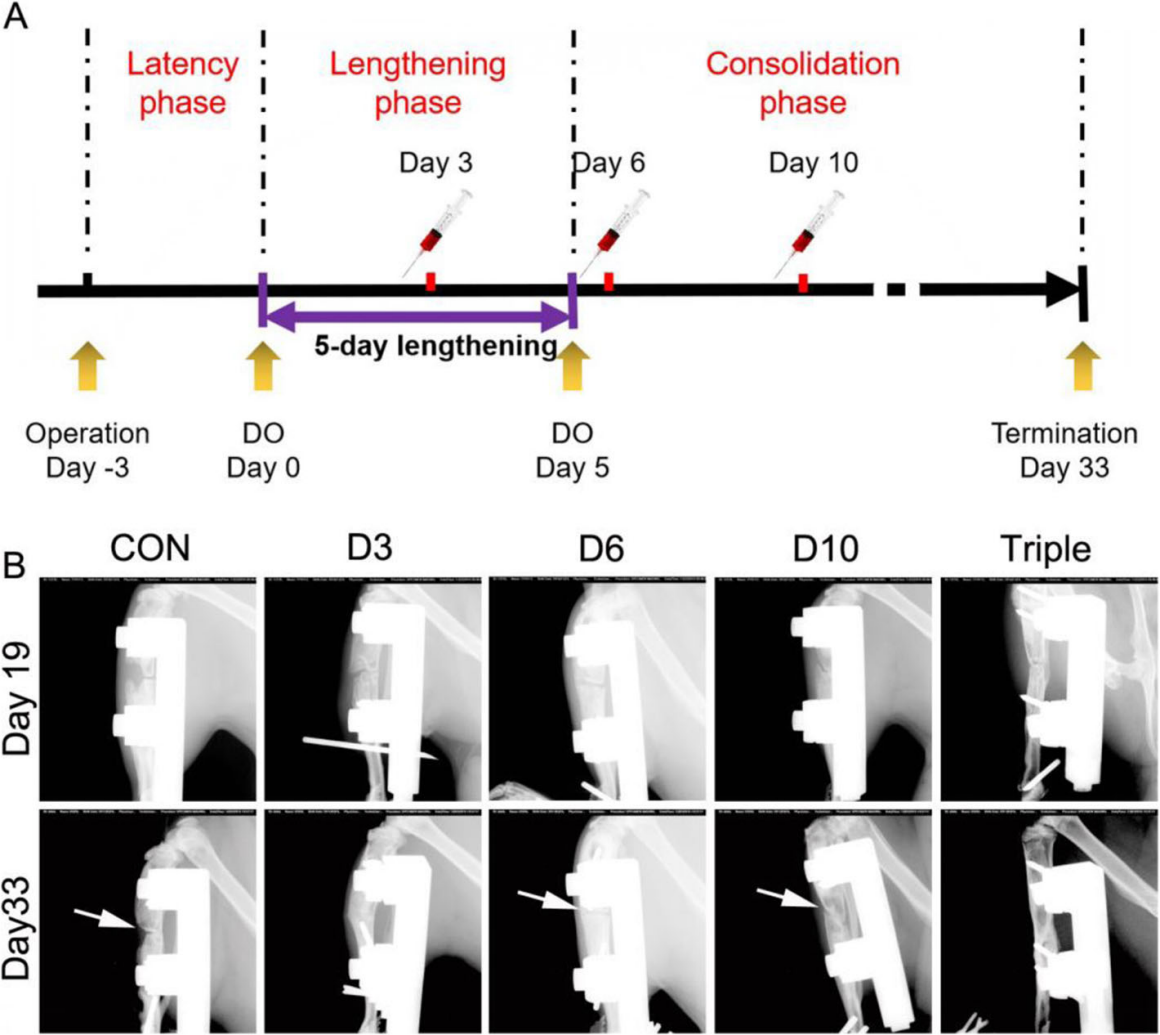
Animal experiment design and dynamic imaging changes of distraction regenerates in the DO animals. Animals were treated with single injection of MSCs on day 3 (D3), day 6 (D6), or day 10 (D10), or one injection of MSCs on day 3, day 6, and day 10 (triple). **a** Schematic timeline of cell injection in animals. **b** Series X-ray images showed the dynamic changes of bone healing after 19 (day 19) or 33 days (day 33) of lengthening. White arrows point to the existing bone defect gaps after 33 days of lengthening began

We rationalized that the dynamic changes in the ratio of circulating MSCs and SDF-1 may be a good indicator for the determination of possible time points of cell therapy in DO. Then, we designed the animal experiment according to the results of circulating MSCs and SDF-1. The schematic diagram shows the study timeline of the animal experiments (Fig. 2a). The rats were subjected to a single injection of PBS as controls (CON), a single injection of MSCs on day 3 (D3), day 6 (D6), or day 10 (D10) after the lengthening began, or one injection of MSCs on day 3, day 6, and day 10 (triple) after the lengthening began, respectively (Fig. 2a). Sequential X-ray images showed that bone defect gaps existed in all the groups on day 19 after the lengthening began (Fig. 2b). The bone defect gaps were totally bridged in the groups treated with single injection of MSCs at lengthening phase (day 3) or triple injections on days 3, 6, and 10 after 33 days (Fig. 2b). However, the defect gaps were still presented in the animals treated with PBS or single injection of MSCs on day 6 or day 10 after lengthening began (Fig. 2b).

### Three-dimensional (3D) microstructure of bone regenerates

**Fig. 3:**
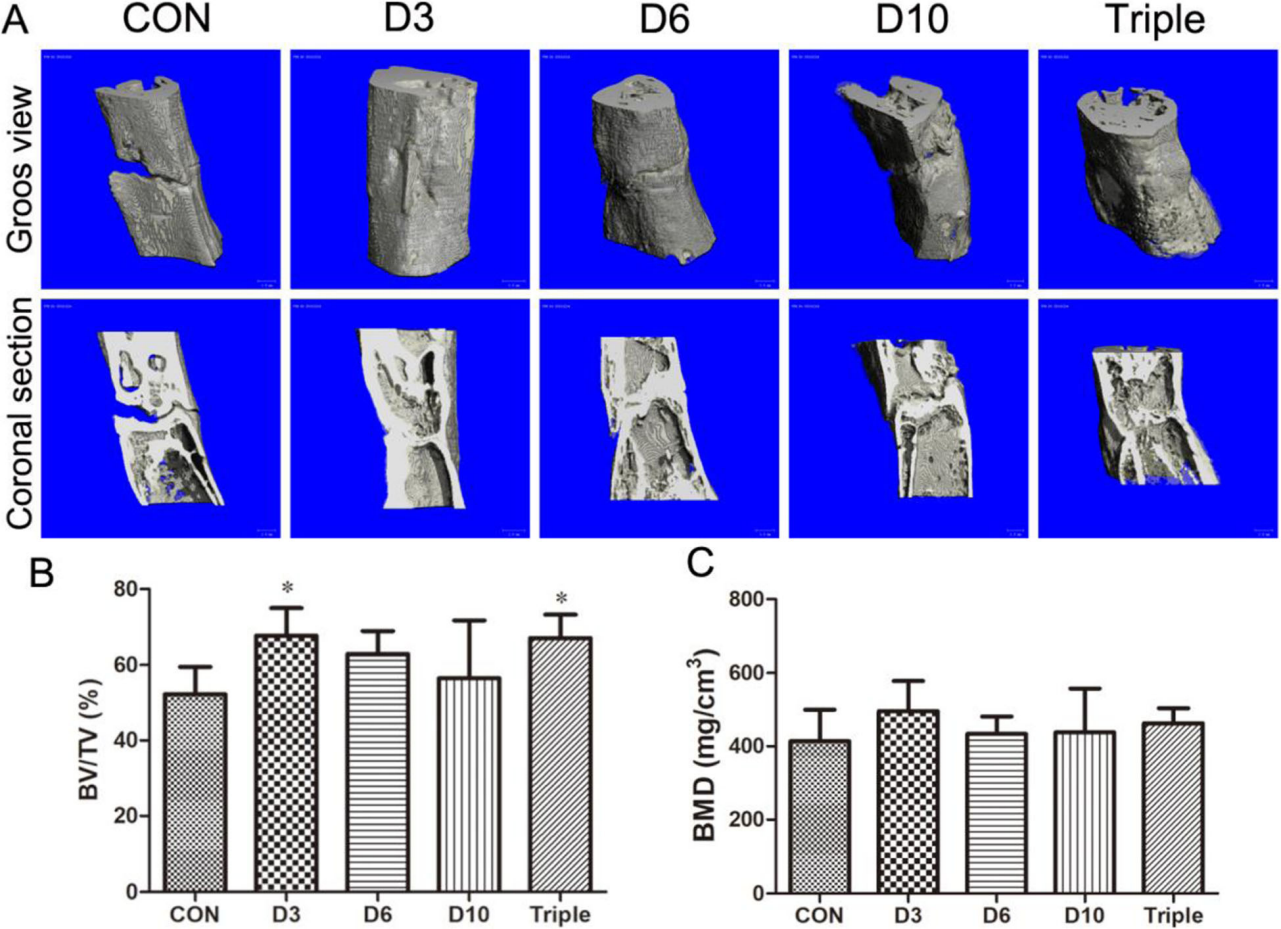
Microstructural changes of the distraction regenerate measured by Micro-CT in the DO animals. Animals were treated with single injection of MSCs on day 3 (D3), day 6 (D6), or day 10 (D10), or one injection of MSCs on day 3, day 6, and day 10 (triple). **a** 3D images of entire or coronal section of the distraction regenerates. **b**, **c** Quantitative results of micro-CT analysis including bone volume ratio (BV/TV) and bone mineral density (BMD). Data were shown as mean ± SD (*n* = 8). *p < 0.05 vs. control

On day 33 after lengthening began, the samples were harvested for ex vivo assessments. From the 3D reconstruction of micro-CT images, we observed the obvious defect gaps in the distraction regenerates of the PBS treated control group on day 33 after lengthening began (Fig. 3a). Bone defect gaps were also observed at the center of the regenerates in the day 6 or day 10 single-injection group (Fig. 3a). The results indicated that the bone consolidation of the regenerates was not complete in the three groups above. However, the bone defect gaps were totally bridged in the day 3 single-injection group or triple-injection group, with significant increases in BV/TV (29.5%, *p* < 0.05, or 28.3%, *p* < 0.05) when compared to the control group (Fig. 3a, b), indicating their bone consolidation was much better than the control group. Although BMD values of the distraction regenerates showed a similar trend as BV/TV, no significant difference among the groups was found (Fig. 3c).

### Mechanical properties of bone regenerates

**Fig. 4:**
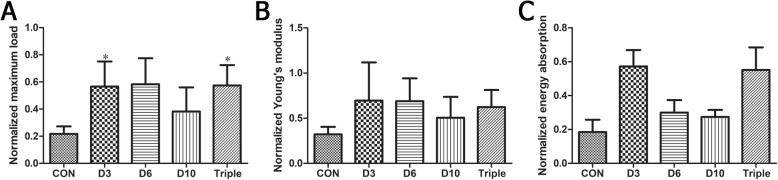
Mechanical properties including maximum load, Young’s modulus, and energy absorption of the affected tibial normalized to the contralateral tibiae in the DO animals. Animals were treated with single injection of MSCs on day 3 (D3), day 6 (D6), or day 10 (D10), or one injection of MSCs on day 3, day 6, and day 10 (triple). Data were shown as mean ± SD (*n* = 8). *p < 0.05 vs. control

Four-point bending test was performed to analyze the bone strength. Normalized maximum loading, Young’s modulus, and energy absorption were increased by 158.2% (*p* < 0.05), 93.5% (*p* > 0.05), and 190.4% (*p* > 0.05) in the day 3 single-injection group compared to the control group treated with PBS (Fig. 4). The mechanical property of the regenerate tissue in the animals that received 3 injections of MSCs was also enhanced when compared with the controls, with the maximum load remarkably increased by 167.0% (*p* < 0.05) (Fig. 4). However, there was no significant change in the mechanical properties of the animals that received single injection on day 6 or day 10 after the lengthening began (Fig. 4).

### Histological assessments in decalcified samples

**Fig. 5:**
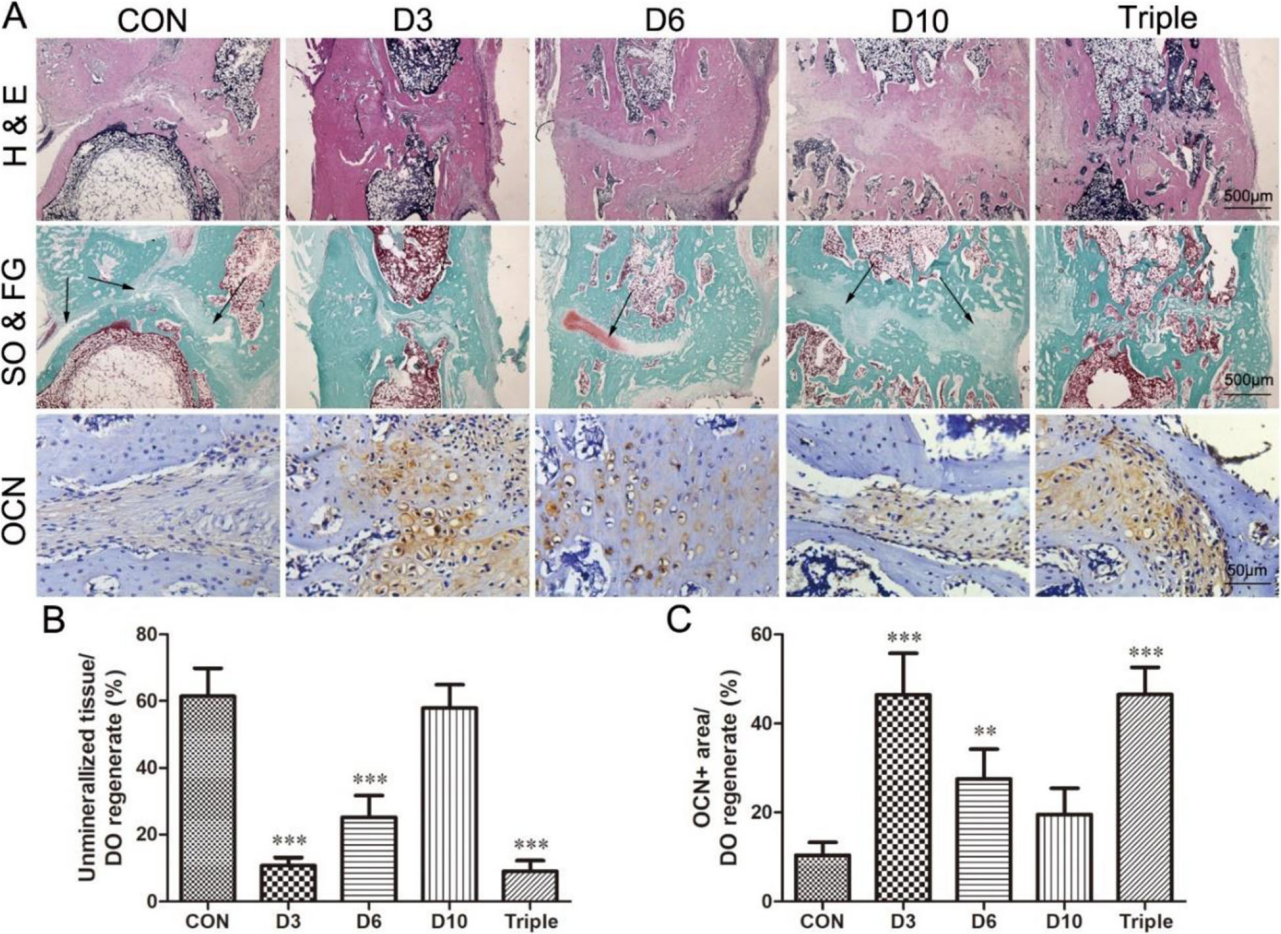
Representative histological images and the quantitative results of the distraction regenerate in the DO animals. Animals were treated with single injection of MSCs on day 3 (D3), day 6 (D6), or day 10 (D10), or one injection of MSCs on day 3, day 6, and day 10 (triple). **a** Samples were stained with H&E, Safranin O & Fast Green (SO & FG), or immunohistochemical staining with osteocalcin expression (OCN). Arrows indicate the unmineralized tissue. Data were shown as mean ± SD (*n* = 8). **b** Quantitative results of unmineralized tissue per DO regenerate. **c** Quantitative results of OCN-positive expressing tissue area per DO regenerate. *p < 0.05, **p < 0.01, ***p < 0.001 vs. CON

H&E and Safranin O & Fast Green stains were used to assess bone repair of samples. The ratio of unmineralized tissue in the regenerate was also determined by the Safranin O & Fast Green stain. The results showed that unmineralized fibrous tissues still presented in the distraction regenerate in the control group or day 10 treatment group (Fig. 5a). Cartilaginous tissues were also found in most of the samples of the days 3 and 6 and triple-injection group (Fig. 5a). Much less unmineralized tissue with continuous cortical bone were observed in the day 3 (− 50.6%, *p* < 0.001) and triple-injection group (− 52.3%, *p* < 0.001) compared with the control group (Fig. 5a, b), indicating advanced bone consolidation in the two groups. The result of immunohistochemistry showed that the expression of bone formation marker osteocalcin (OCN) was significantly increased in the regenerates of the day 3 (36.0%, *p* < 0.001), day 6 (17.1%, *p* < 0.01), and triple-injection group (36.1%, *p* < 0.001) compared to the control group, suggesting enhanced bone formation (Fig. 5). In this study, the GFP-expressing cells were not observed after 33 days of lengthening in the animals, indicating that the injected cells may not directly incorporate into the regenerates (Supplementary figure1).

### Histomorphometry in non-decalcified samples

**Fig. 6:**
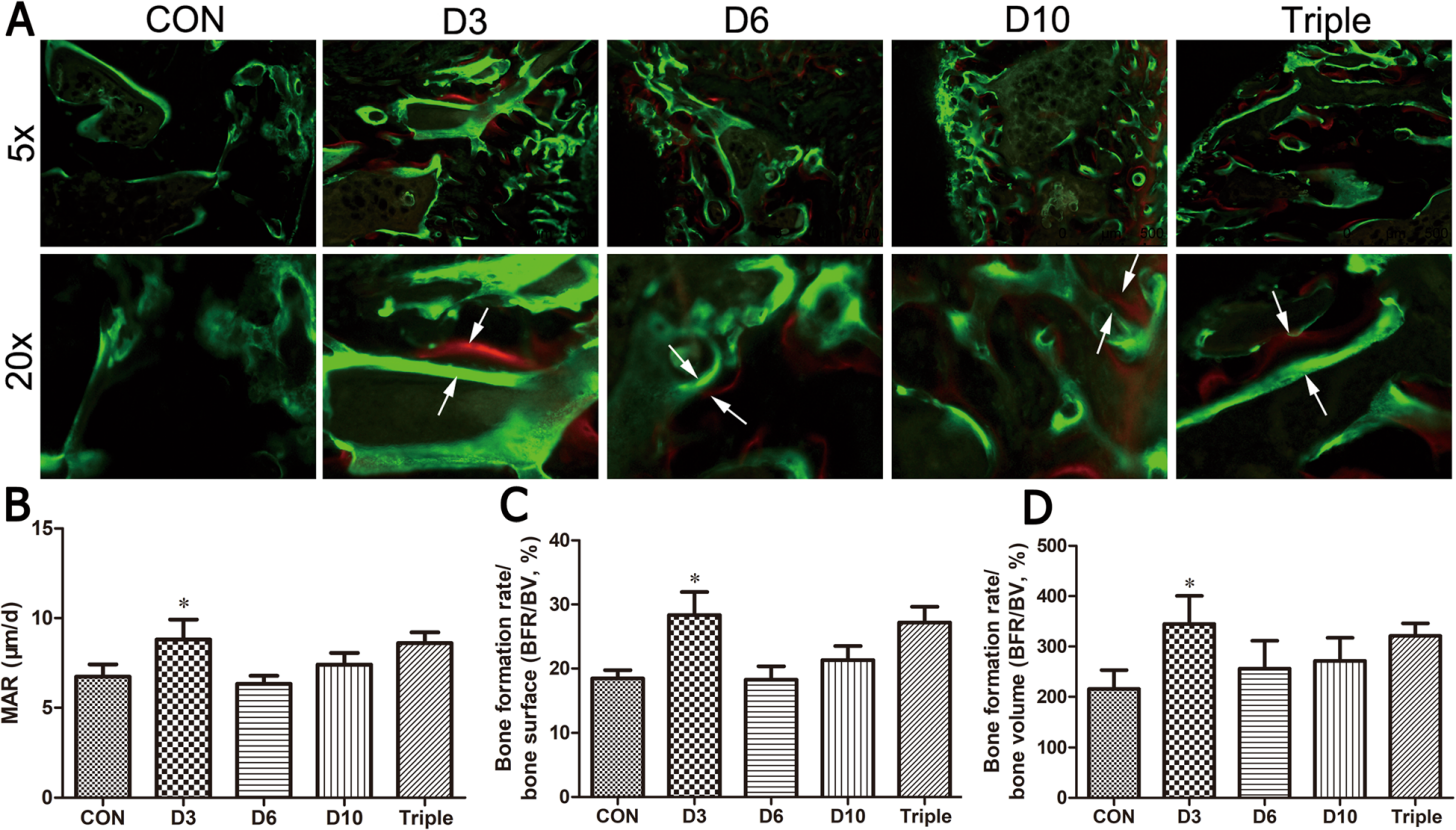
Dynamic bone formation in the distraction regenerate measured by histomorphometry in the DO animals. Animals were treated with single injection of MSCs on day 3 (D3), day 6 (D6), or day 10 (D10), or one injection of MSCs on day 3, day 6, and day 10 (triple). **a** In vivo double labelling in the distraction regenerate tissue. White arrows indicate the double labels of bone formation between 10 days. **b**–**d** Quantitative bone formation parameters including mineral apposition rate (MAR, **b**), bone formation rate per unit of bone surface (BFR/BS, **c**), and bone formation rate of bone volume (BFR/BV, **d**)

The undecalcified samples were used to determine the dynamic bone formation in the distraction regenerates by histomorphometry (Fig. 6). The distance between green (calcein) and red (xylenol orange) dyes represented the rate of new bone formation (Fig. 6a). The results show that the fastest bone regeneration rate was found in the day 3 injection group, as evidenced by the higher MAR (30.7%, *p* < 0.05), BFR/BS (53.6%, *p* < 0.05), and BFR/BV (59.8%, *p* < 0.05) when compared to the control group (Fig. 6b–d). The bone regeneration rate was also slightly increased in the triple-injection group, with no significant change compared to the control group.

## Discussion

In the first part of this study, we found the ratio of circulating MSCs to peripheral mononuclear cells as well as the serum levels of SDF-1 were gradually increased from the day of operation, and then reached to their peaks on day 3 after bone lengthening began, then decreased gradually afterwards. The results indicated that traumatic osteotomy and bone lengthening may have enhanced the migration of MSCs and upregulated the serum levels of pro-migration cytokines. Inflammatory cytokines such as IL-1, IL-6, and TNF-α involve in fracture repair and play an important role in initiating the repair cascade following injury [12, 21]. They induce downstream responses to injury through recruiting inflammatory cells, enhancing extracellular matrix synthesis, and stimulating angiogenesis [22]. These cytokines are produced immediately after injury and lasted for a limited time period [12]. Like those in fracture repair, the expression of pro-inflammatory cytokines IL-1 and IL-6 would be upregulated after osteotomy and then returned to baseline levels rapidly in the latency phase [12, 21]. The expression of IL-6 was elevated for a second time, once lengthening was initiated and when the mechanical strain was applied to the callus. However, in our current study, we have not observed such significant changes in IL-1β. SDF-1 is an important chemokine binding to its receptor CXCR4, which then regulates stem cell trafficking to the ischemic area, such as callus, and induces their subsequent differentiation [23, 24]. Our results indicated that SDF-1 may involve in the systemic migration of MSCs during DO as indicated by their similar dynamic changes. It is not clear if the circulating MSCs would home to the injury site and involve in the bone healing during DO. Our previous study revealed the important role of circulating stem cells in promoting fracture healing. Circulating MSCs can migrate and home to the bone injury site [13, 14]. However, literatures also show that periosteum is the main source of stem cells for bone healing [25, 26]. A very recent study showed that Mx1+aSMA+ periosteal stem cells could rapidly migrate towards the fracture site and supply osteoblasts and chondrocytes and recover new periosteum [26]. Evidence from another study also suggests that injury can introduce plasticity or interconversion between periosteal stem cells and bone marrow-derived MSCs [25]. We believed that both of circulating MSCs and periosteum are essential for bone healing, especially for large bone defect healing. The recruitment of circulating MSCs may promote bone defect healing when the periosteum is missing at the defect site. In our study, the dynamic changes in the ratio of circulating MSCs as well as the expression levels of SDF-1 provided valuable information to determine the possible time points for cell therapy in DO. There are abundant literatures on experimental animal work documented the potential application of marrow derived MSCs to enhance bone formation [11]. However, little is known about the role of MSCs in DO. Recently, Ransom and his colleagues suggested that tissue-resident stem cells or progenitor cells are mechanically responsive [27]. Mechanotransduction via focal adhesion kinase (FAK) in skeletal stem cells during DO activates the gene regulatory network. And they also found that the skeletal stem cells that reside in craniofacial tissue may arise from primitive neural crest cells during development [27]. When the lengthening phase activates, the MSCs would differentiate into osteoblastic cells or chondrocytes [12]. However, whether skeletal stem cells paly a similar role in limb lengthening as that in the craniofacial bone lengthening remains unknown.

Several studies of MSC-based therapy have tried to inject MSCs at different time points during DO but had inconsistent results [11]. For example, Guevara et al. reported a negative effect by injecting MSCs at 24 h after fracture surgery [28]. The authors suspected that animal model of bone injury may be not challenging enough to discriminate any augmentation provided by stem cells. On the other hand, some studies did show positive results by injecting MSCs at early time points before lengthening phase [29, 30]. In fact, more and more studies have reported that injecting MSCs in or after the lengthening phase showed beneficial effects on bone formation and consolidation [31–34].

In the second part of this study, we found that administration of MSCs on day 3 after bone lengthening, an early time point, achieved an enhanced effect on bone consolidation. In these animals, mechanically robust bone regenerate exhibited enhanced bone formation and remodeling in the regenerates. Similar results were also found in the animals that received triple injections of MSCs at three time points. However, when we injected MSCs at later time points (day 6 or 10), the healing effect was diminished. We proposed that the different healing effects may be due to the various molecular and cellular responses when MSCs were given at three different phases of DO [12, 35]. MSCs would be recruited from the latency phase, and they then differentiate into osteoblasts or chondrocytes in the lengthening and consolidation phase [12, 35]. When the level of circulating MSCs or cytokines reaches to its peak, it may indicate more MSCs are being recruited to the injury sites and actively participated in the healing processes. However, MSCs become less active in the consolidation phase, and only osteoblastic cells may continue to secrete mineral matrix. When MSCs were injected locally at the consolidation phase, they may not differentiate into osteoblasts as they may do in the lengthening phase. In the triple-injection group, however, the effect of MSCs injection was similar to that of the day 3 group, indicating that MSC-based cell therapy performed at the lengthening phase (early time point) is more effective and further administration of MSCs at consolidation phase (later time point) does not have any additive effects. Despite the positive findings, the roles of circulating MSCs in bone healing during DO are still to be determined. The injected GFP-expressing cells may only exert paracrine effects in promoting bone healing but not directly engrafted in bone formation. More and more evidences showed that trophic factors including growth factors and/or microRNAs secreted by MSCs may have dominant effect on tissue regeneration [36, 37]. Trophic activities of MSCs, either resident or introduced exogenously, may be further fine-tuned via mechanical stimulation in DO. However, mechanisms as why early application of MSCs augmented bone formation is still unclear. Previous evidence showed that growth factors, such as BMP2, BMP7, and VEGF, as well as their receptors, essential for bone and blood vessels formation, are highly expressed in the regenerated tissue during bone lengthening phase but gradually decreased at the consolidation phase [12, 38]. Hence, the trophic effect of MSCs may be mediated by their secreted growth factors, which become more effective at the lengthening phase, but less effective at the consolidation phase in DO. Our previous study has already showed the beneficial effect of secretome derived from human fetal stem cells on bone consolidation in DO [15]. However, the components of secretome were still undetermined, and the multiple injections of secretome may limit its application. MSC-based therapy may have the advantage of sustainable releasing of growth factors which may avoid repeatedly injecting of the bioactive factors.

## Conclusion

In summary, we have demonstrated that single administration of MSCs locally into the distraction regenerate when the serum level SDF-1 and ratio of circulating MSCs reaching to the highest level at lengthening phase could enhance early bone consolidation in the rat DO model. Current findings suggested that day 3 after the bone lengthening began may be the optimal time point for MSCs therapy to promote early bone consolidation in distraction osteogenesis, indicating a valuable clinical implication for applying stem cell therapy in DO patients with poor healing outcomes.

## Supplementary information

**Additional file 1: Figure S1.** The expression of green fluorescent protein (GFP) in the distraction regenerates in the DO animals. Animals were treated with single injection of MSCs on day 3 (D3), day 6 (D6), or day 10 (D10), or one injection of MSCs on day 3, day 6, and day 10 (Triple). Data showed no positive expression of GFP signal in the regenerate after 33 days of lengthening, indicating that the injected cells may not directly incorporate into the regenerates. Scale bar: 100 μm.

## Data Availability

All data generated and/or analyzed during this study are included in this manuscript and its supplementary information files.

## Abbreviations

BFR/BS: Bone formation rate per unit of bone surface
BFR/BV: Bone formation rate of bone volume
BMD: Bone mineral density
BV: Bone volume
BV/TV: Ratio of bone volume to total tissue volume
CT: Computed tomography
dL.S: Double-labeled surface
DO: Distraction osteogenesis
GFP: Green fluorescent protein
IL-1β: Interleukin 1β
MAR: Mineral apposition rate
MS/BS: Ratio of mineralizing surface to bone surface
MSCs: Mesenchymal stem cells
SDF-1: Stromal cell-derived factor 1
sL.S: Single-labeled surface
TV: Tissue volume
