# Supplementary material for: Administration of allogeneic mesenchymal stem cells in lengthening phase accelerates early bone consolidation in rat distraction osteogenesis model
Source: Stem Cell Res Ther. 2020 Mar 20;11:129. doi: 10.1186/s13287-020-01635-5 (PMC7083044; doi:10.1186/s13287-020-01635-5)
Supplement: Supplementary file 1 — Additional file 1: Figure S1. The expression of green fluorescent protein (GFP) in the distraction regenerates in the DO animals. Animals were treated with single injection of MSCs on day 3 (D3), day 6 (D6), or day 10 (D10), or one injection of MSCs on day 3, day 6, and day 10 (Triple). Data showed no positive expression of GFP signal in the regenerate after 33 days of lengthening, indicating that the injected cells may not directly incorporate into the regenerates. Scale bar: 100 μm. [file 13287_2020_1635_MOESM1_ESM.docx]

Supplementary figure


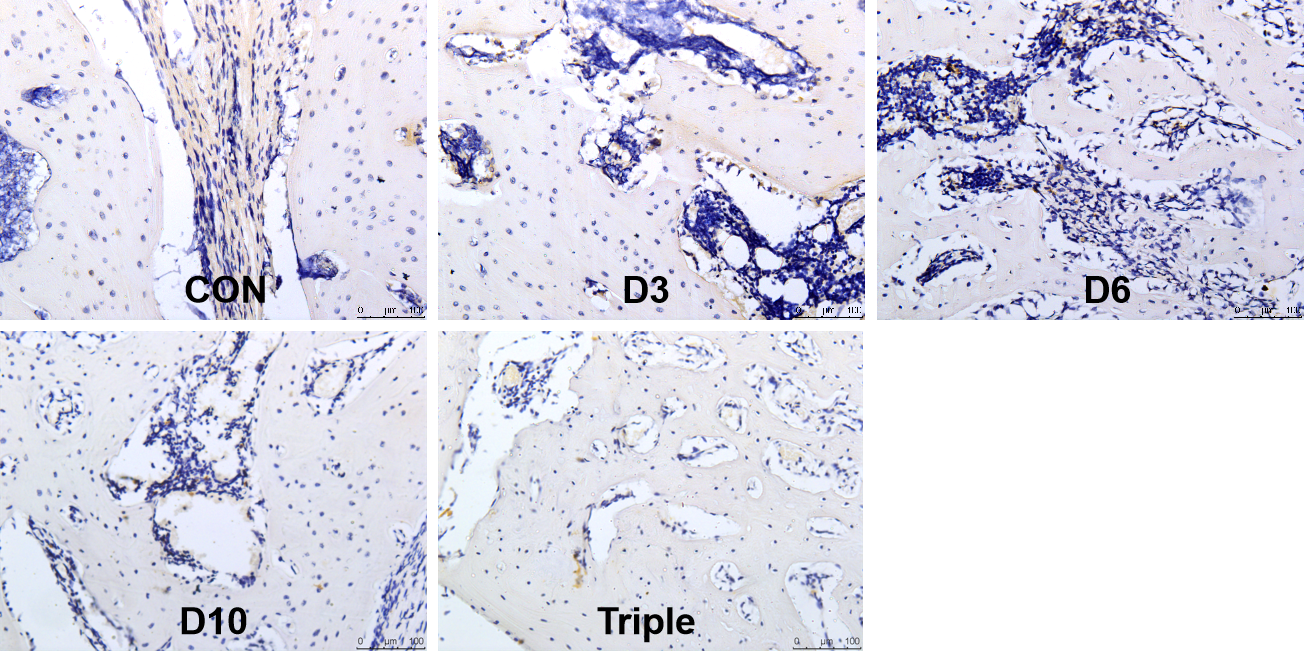


**Supplementary figure 1.** The expression of green fluorescent protein (GFP) in the distraction regenerates in the DO animals. Animals were treated with single injection of MSCs on day 3 (D3), day 6 (D6), or day 10 (D10), or three injections of MSCs on day 3, day 6, and day 10 (Triple). Data showed no positive expression of GFP signal in the regenerate after 33 days of lengthening, indicating that the injected cells may not directly incorporate into the regenerates. Scale bar: 100 μm
